# NGO Partnerships in Using Ecotourism for Conservation: Systematic Review and Meta-Analysis

**DOI:** 10.1371/journal.pone.0166919

**Published:** 2016-11-28

**Authors:** Tania P. Romero-Brito, Ralf C. Buckley, Jason Byrne

**Affiliations:** 1 Griffith School of Environment, Griffith University, Gold Coast, Queensland, Australia; 2 International Chair in Ecotourism Research, Griffith University, Gold Coast, Queensland, Australia; University of Tasmania, AUSTRALIA

## Abstract

We analyse 214 cases worldwide where non-governmental organisations (NGOs) use ecotourism for conservation. Other stakeholders in these initiatives include local communities, the private sector, and government agencies. Stakeholder relationships determine NGO roles and project management structures and governance. We classified cases into 10 structural categories based on the initiating stakeholder and the NGO role, and used these categories to analyze geographic patterns and success factors. Most of the 214 cases are community-based (~170; 79%); most are in developing countries (190; 89%); and most are in protected areas (196; 91%). Frequencies of structural categories differ between continents. More cases in Latin America and Asia are initiated by NGOs and local communities, and more in Africa by the private sector. Case-study authors used a range of economic, socio-cultural and environmental criteria to judge whether projects were successful. At global scale, we found no significant association between project success and the involvement of private tourism entrepreneurs. Projects involving either local or international NGOs had higher success rates than those that involved both simultaneously. Future research could adopt political ecology approaches to examine: the factors that lead NGOs to adopt ecotourism enterprises; their internal decision-making processes and strategies; their interactions with the stakeholders involved; and their conservation goals and outcomes.

## 1. Introduction

The conservation of biological diversity is urgent, insufficient, and underfunded [1–12]. Practical conservation involves social as well as natural sciences, and multiple stakeholders including private, community and non-government organisations (NGOs) as well as government and multilateral agencies [13–19]. Amongst these stakeholders, NGOs are assuming an increasingly important role, through: formal and informal political lobbying; buying or covenanting land for conservation; and forming partnerships to undertake specific conservation projects [19–30]. Establishing conservation-oriented ecotourism enterprises is one such approach. These enterprises aim to switch land and resource use from consumption to conservation, and to resist external threats such as large-scale land use change associated with primary industries [31–33]. This approach requires that non-profit NGOs, and their project partners, must operate within the competitive commercial tourism sector.

Relationship between stakeholders are often complex, and partnership arrangements influence governance, management and outcomes of projects [29, 30, 34, 35]. NGOs use different approaches and strategies, with different outcomes, in developed and developing countries respectively [31, 32, 36–47]. Some analysts have argued that NGOs have successfully used ecotourism as a local conservation tool, either by operating tours, influencing the management of protected areas, or raising local awareness [24, 32, 33, 36, 43, 48–53]. Other authors, however, have concluded that NGOs may be driven more by global discourses, such as those related to poverty alleviation, development, conservation, equity, and the role of non-state organisations; and that they may prioritize their own agendas over local communities and conservation needs [54–57]. Overall, it appears that NGO actions and strategies are determined partly by historical, environmental and legislative contexts in the countries, cultures and communities concerned; and partly by the internal history and structure of each NGO as an organization [25–28, 31, 32, 40, 42, 46, 58–60].

Many authors, for example, have suggested that NGOs in developed countries preferentially operate ecotourism enterprises themselves, or form partnerships with government agencies in tourism policy and management for protected areas [24, 40–42, 49, 51–53]. In developing countries, in contrast, ecotourism approaches by NGOs commonly combine nature conservation and poverty alleviation, often in challenging political circumstances. Tactics include the establishment of community-based ecotourism projects [22, 32, 59, 60], and campaigns to promote social awareness or create social pressure to halt unsustainable developments [33, 36, 43, 50, 56, 58, 61].

None of these previous studies have conducted a global comparative analysis of approaches and outcomes at the scale of individual case studies. Here, therefore, we present a systematic worldwide review of published NGO-based ecotourism projects. The overall question addressed is: where, how, and how well do these projects operate? Specific questions examined are as follows. 1. How many NGO-based ecotourism projects are currently operational worldwide? 2. Where do they operate? 3. What stakeholders are involved? 4. Which stakeholder initiated each project? 5. What is the role of the NGO in each case? 6. What internal governance structure has each adopted to manage interactions between stakeholders? 7. How successful, or otherwise, has each project proved? 8. What specific environmental, social and economic outcomes have they achieved? 9. What factors have contributed to the success or failure of each? 10. What lessons can be learned for future NGO-based ecotourism projects?

We also test three specific predictions derived from previous published literature, as follows. Prediction 1. There are continental-scale patterns in the design, structure and operational parameters of ecotourism projects, creating “regional signatures” which reflect the different histories of land tenure and management, and past tourism patterns such as safaris in Africa and outfitters in North America (37). Prediction 2. The proportion of ecotourism projects which are successful is higher for those which are initiated or controlled by a private sector partner, because of business experience and commercial connections with the tourism industry [23, 34, 60, 62–65]. Prediction 3. The proportion of ecotourism projects which are successful is higher for those that include both international and local-scale NGOs, since international NGOs can generally mobilise more funds, broader expertise, and greater political power, whereas local NGOs generally have greater knowledge of local circumstances, and better connections and rapport with local communities and other stakeholders [32, 66–70].

## 2. Methods

### 2.1 PRISMA Protocol and additional analyses

**Fig 1:**
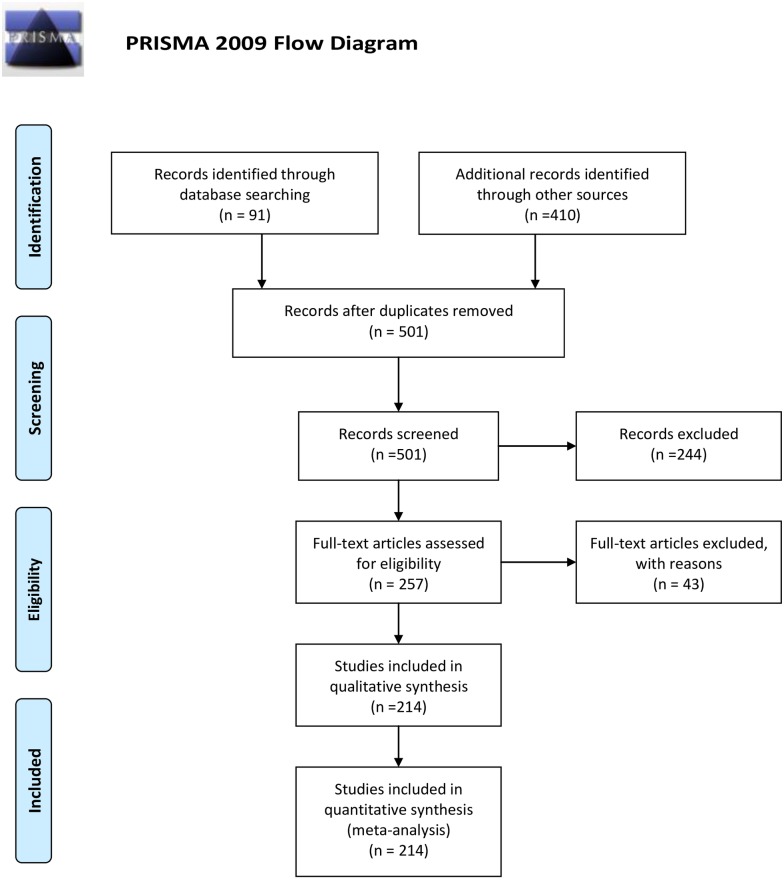
PRISMA Flowchart. *From*: Moher D, Liberati A, Tetzlaff J, Altman DG, The PRISMA Group (2009). *P*referred *R*eporting *I*terns for *S*ystematic Reviews and *M*eta-*A*nalyses: The PRISMA Statement. PLoS Med 6(7): e1000097. doi:10.1371/journal.pmed1000097 For more information, visit www.prisma-statement.org.

We adopted the standard requirements of systematic reviews as specified by PRISMA [71]. Our methods are fully described below, and there is no additional methodological protocol [72]. The PRISMA checklist is attached (S1 Table), and the flowchart is provided as Fig 1. Under item 16 of the PRISMA protocol, Additional Analyses, we added three components as below, relating respectively to sources, data and analysis.

Regarding sources, the PRISMA protocols provide for a series of successive screening steps, but then treat all selected publications as of equal weight. Here, however, we distinguished three sets of sources, providing different levels of detail, and hence contributing differentially to each individual research question as outlined above. Regarding data, the PRISMA protocols are designed primarily for quantitative data, whereas we included both quantitative and qualitative information, derived from analysis of text. Regarding analysis, the PRISMA protocols assume a single-stage analysis of the final set of publications which survive the screening processes. In this analysis, however, we adopted a two-stage approach. First, we used qualitative data, extracted from some of the sources, to generate a new structural classification of cases. This classification constitutes the first result of our analysis. We then used the categories in this classification as an additional parameter for quantitative analysis of cases from all sources.

### 2.2 Searches, sources and information extracted

Our sources were derived as follows. We searched the refereed published literature between May 2012 and June 2015 using standard academic search engines including Web of Science^®^, Google Scholar^®^ and Science Direct^®^. We used search terms ‘ecotourism’, ‘community-based tourism’, ‘conservation tourism’, ‘sustainable tourism’, ‘venture tourism’, ‘voluntourism’ and ‘cultural tourism’, in Boolean combination with ‘non-governmental organisation’, ‘civil association’, and ‘non-profit organisation’. We tracked citations both forwards and backwards.

We included only peer-reviewed journal articles, books, and book chapters, published in English or Spanish. We examined the geographic distribution of the studies identified, as an outcome-level check against possible bias through language of publication, and we examined the academic journals and disciplines where each case study was published, as an outcome level check against possible bias associated with different academic disciplines.

Our data were extracted as follows. For each individually identifiable case study, we extracted information on: (i) the specified objectives of each study; (ii) the research methodologies adopted; (iii) the principal country concerned; (iv) whether or not each ecotourism initiative took place within a protected area; (v) the declared aims and implementation date of each project; (vi) the type and scale of the NGOs involved; (vii) any other stakeholders involved, and their roles; (viii) the NGO goals, roles and rationales for adopting ecotourism strategies; (ix) the specific outcomes of each project as described by each study’s authors; and (x) whether or not these authors considered each project to be successful, and why. Most of these data are thus qualitative, categorical or dichotomous. We examined frequencies of different categories as an outcome-level check against possible bias towards reporting only projects considered successful.

### 2.3 Coding, compression and categorisation

Most of the cases considered in this meta-analysis were published in social science journals, as outlined below in the first section of the Results. They presented their objectives and methods (information types (i) and (ii) in the preceding paragraph), and also their findings, in textual rather than numerical form. To conduct this review, we therefore used a combination of qualitative and quantitative approaches, to simplify the data structure sufficiently for statistical analysis, without losing the richer degree of detail contained in text presentations.

Qualitative analysis extracts distinct and identifiable concepts from a set of text, and iteratively builds a hierarchical pyramid of constructs. This approach requires that all the text analysed addresses the same issues, so that the lowest-level concepts can be linked into higher-level constructs. Of the ten sets of data extracted from each case analysed here, some represent logically linked concepts, which can be incorporated in the same qualitative analysis. Others, however, are logically independent of each other, so their associations can only be considered in a statistical sense. For example, there may be a statistical association between the success or failure of each project, data item (x) above, and the country where it took place, item (iii), but there is no logical qualitative hierarchy between them.

To conduct statistical meta-analysis of patterns, we therefore converted coded concepts, which are philosophically part of a qualitative analytical approach, to statistical categories, which are philosophically part of a quantitative approach. For some of the types of information extracted from each case, this conversion is straightforward. For example, text specifying where each case study was conducted can be coded as inside or outside a protected area, and this can be converted unambiguously to a dichotomous variable. Similarly, location text can be coded as developed or developing nation, which converts unambiguously to a second dichotomous variable. It can also be coded as a country name, which converts unambiguously to a categorical “continent” variable. Conversion from country to continent is unambiguous, but involves substantial data compression.

For information of types (v) to (vii) above, we adopted a combined data compression approach, using several sets of case-study information simultaneously in order to generate a categorical classification of case studies. The details are set out in the next section. This created a new, derived, categorical variable for each case study, which we refer to as structural classification type, and which we then used in subsequent statistical analysis. We also used a qualitative approach to identify the various rationales used by NGOs for adopting ecotourism as a conservation tool, item (viii) above.

Finally, for information of types (ix) and (x) above, we used a qualitative approach to identify how individual case-study authors described case-study outcomes, and how they judged the success or failure of each of the projects concerned. We also converted the highest-level construct for item (x), project success or failure as judged by case-study authors, to a dichotomous variable for use in statistical analysis. We did not attempt to re-interpret the judgements of case-study authors, firstly since we did not have their direct experience of the projects concerned, and secondly since the various published studies did not all have the same aims or employ the same criteria.

### 2.4 Structural classification

We developed the structural classification through standard iterative qualitative analysis of text [73, 74], obtained by searching each source publication for information on how projects were established, structured, developed and operated. We used this information to identify the stakeholders involved, the roles adopted by each, and the ways in which each project was initiated, controlled, governed and managed. This yielded a matrix of [cases] x [stakeholder roles and interactions]. We then simplified the structure of this information set iteratively, by simultaneously grouping and re-grouping both the cases and the stakeholder parameters, so as to generate a classification of cases into structural categories. We continued this process until no further amalgamations were possible without loss of significant information discriminating between categories. This classification was not available *a priori*, but is derived as part of our analysis, and is therefore presented below in the Results.

This process yielded 10 structural categories, with distinctions between categories reflecting only two of the case parameters: the stakeholder that initiated the project, and the role adopted by the NGO. We were therefore able to define each category using fixed combinations of those two factual criteria. That is, we initially derived or identified the categories using an iterative qualitative procedure, which involves ambiguities; but we ultimately defined the categories using objective criteria, which are unambiguous. These combinations and definitions are also derived as part of our analyses, and are therefore presented below in the Results.

We assigned each of the individual cases to one of these structural categories, and used those categories as an additional parameter for further analyses. For 37% of cases, the allocation of cases to categories was carried out independently by the first two authors, and then compared so as to calculate an inter-coder reliability rate, ICRR. Since the final criteria used to define the categories are unambiguous, the ICRR for these cases was 100%.

### 2.5 Statistical tests

To address the questions predictions outlined earlier, we tested for associations between the categorical variables for each case study, including the additional variable derived from the structural classification. We used Fisher’s Exact Test for all tests of associations, two-tailed except where otherwise stated. Since all the parameters are categorical, parametric statistical techniques are not applicable.

## 3. Results

### 3.1 Presentation of findings

We present our findings under seven headings, as follows. (i) Summary of data sources and screening using a PRISMA flowchart. (ii) Structural classification of cases, based on stakeholder involvement and roles adopted, and project initiation and governance. (iii) Rationales advanced by NGOs for taking part in ecotourism projects. (iv) Social, economic and environmental outcomes of each case study, and the mechanisms by which these outcomes were achieved, focussing on quantifiable conservation outcomes where these are available. (v) Criteria used by case study authors to evaluate success or failure of each project, and the underlying reasons where provided. (vi) Country-by-country geographic distribution of case studies, and test for associations with protected areas, level of development, and structural category. (vii). Associations between success or failure, and other variables.

### 3.2 Sources and screening

We identified source materials in three categories. The first category (S2 Table) comprises 34 refereed journal articles and books, which do not present detailed information on individual cases, but provide critical context for detailed meta-analysis of case studies [22–25, 27–36, 40–43, 46, 48–60, 65]. The second category (S3 Table) consists of 50 refereed journal articles and books, which each focus specifically on one or more individual case studies, 57 cases in all [26, 44, 45, 64, 75–121]. The third category comprises three books which compile previously published case studies in ecotourism [37, 38, 47]. These compendia include ~410 individual cases in total, of which 200 (48%) involve NGOs, and 157 include sufficient information to be incorporated in this analysis (S4 Table). Combining all three sources, we identified 257 cases in all, of which 214 cases were usable. The PRISMA flowchart for this process is provided as Fig 1.

The journal articles and books analysed were published in three main disciplines: tourism (55 articles, 65.4%), ecology (9 articles, 10.7%), and geography (4 articles, 4.7%). The rest were published in fields such as conservation and biodiversity, ornithology, forestry, human ecology, policy, social development, interdisciplinary fields, anthropology, rural studies, and education: >40 different journals in all. A wide range of quantitative and qualitative methods were adopted in these studies, including surveys, interviews, participant observation, ethnographic approaches, focus groups, discourse analysis, case studies, content analysis, narrative opinions and mixed approaches.

### 3.3 Structural classification and NGO roles

The iterative qualitative analysis used to derive the structural classification yielded four key simplification steps or insights. The first of these is that the cases can be classified using a matrix of stakeholder type vs stakeholder roles. Four stakeholder types can be distinguished: government agencies, private operators, local communities, and NGOs. The second is that the roles adopted by three of these four stakeholder types are largely consistent across cases, whereas the NGO stakeholders adopt a range of roles which produce different consequences for project structure. Ten NGO roles are distinguishable: lobbyist or promoter, landowner or land manager, champion, ongoing manager, founding manager, certifier, advisor or facilitator, networker, broker, and consultant. The third step is that the single most influential aspect of stakeholder roles, in determining subsequent structure and governance, is which stakeholder originally initiated the project. We therefore reduced the classification to a matrix of initiating-stakeholder vs NGO-role. The fourth and final step is that of the numerous theoretically possible combinations of initiating stakeholder and NGO role, only 10 actually occur in practice. This iterative process thus successfully allocated the 214 individual cases into 10 structural categories, summarised in Table 1. The number of cases differs considerably between categories, from 1 to 63. The intercoder reliability rate was 100% for the 79 cases (37%) classified independently by the first two authors.

**Table 1:** Structural categories.

Category	Initiator	NGO role	NGO actions	No. of cases^a^
1	NGO	Lobbyist / Promoter	Promotes ecotourism through campaigns, code of conducts, protests, marketing, environmental education and policy making	6
2	NGO	Land owner or manager	Manages and safeguards an entire protected area, private or public, and incorporates tourism components	19
3	NGO	Champion	Principal driver of community-based ecotourism (CBET) projects	63
4	NGO	Ongoing manager	Operates its own tours, mostly associated with scientific research for conservation	15
5	NGO	Founding manager	Operates its own tours initially, but intending to hand over management to a local community once established	10
6	NGO	Certifier	Creates certification, awards, and monitoring mechanisms for ecotourism ventures and hotels	1
7	Local community	Advisor / facilitator	Assists and advises in the creation or re-organization of CBET projects.	31
8	Local community	Networker	Links various existing CBET projects together and with other stakeholders, e.g., local government and private tour operators, and/or assists them with promotion strategies. Umbrella organisations.	15
9	Private tour operator	Broker	Supports ecotourism enterprises carried out by private tour operators that supports conservation and social initiatives, and may link them with local communities and/or government	36
10	Government	Consultant	Assists local governments in the design and/or execution of ecotourism projects	7

^a^ n = 203 cases; for 11 cases, the role of the NGO was not clear.

There are six categories where the NGO is the initiator, and four where it is not. In projects initiated by other stakeholders, NGO involvement can occur either at the request of that stakeholder, or through the proactive initiative of the NGO. In categories 1–6, where the NGO is the initiator, NGOs exercise principal control over the project management structure. These categories are distinguished by the role played by the NGO as (1) lobbyist or promoter, (2) land owner or land manager, (3) champion, (4) ongoing manager, (5) founding manager, or (6) certifier. In categories 7–10, projects are initiated and generally also managed by other stakeholders, with NGOs playing secondary roles. In categories 7 and 8, the local community is the initiator. In category 7, the NGO acts as advisor or facilitator. In category 8, the NGO acts as a networker across multiple projects. Category 9 consists of projects initiated by private tour operators, where the NGO acts as a broker to other stakeholders. Category 10 consists of projects initiated by a government agency, where the NGO acts as a consultant.

Globally, the most frequent category (63 cases, 29.4%) is category 3, where the NGO champions its own initiatives. The second most frequent (36, 16.8%) is category 9, where projects are initiated by private-sector partners. The third (31, 14.4%) is category 7, where the NGO acts as advisor and facilitator for projects initiated by local communities. Overall, ~170 of the 214 case studies (~79%) involved community-based ecotourism, but only 46 of these (27%) were initiated by the communities concerned.

Different NGOs preferentially adopt different roles, depending on their profiles, aims and expertise. The role of the NGO can evolve during the course of an individual project: for example, where the NGO acts initially as consultant to a government agency (category 10), but later as champion of the initiative itself (category 3). An NGO may adopt multiple simultaneous roles in the same project: e.g., both as broker (category 9) linking a private operator with local landowners or government, and simultaneously as lobbyist (category 1) for the entire initiative. An NGO may adopt multiple roles in different contemporaneous projects: e.g., as founding manager (category 5) in one project, and advisor or facilitator (category 7) in another.

### 3.4 NGO rationales for adopting ecotourism

From the 57 individual cases described in detail (S3 Table), five initial reasons were identified for NGOs to engage in ecotourism projects. For 14 cases (24.5%), ecotourism projects formed part of a deliberate overarching strategic plan for the NGO concerned, aiming to gain financial and political support for conservation of species and areas. This rationale is most frequent for areas managed directly by the relevant NGO. In six cases (10.5%), the NGO used ecotourism as a conservation strategy to mitigate the environmental impacts of natural disasters such as hurricanes, or external environmental threats such as mining or very large-scale tourism development. In a further six cases (10.5%), the NGO became involved in order to support an ecotourism project initiated by a private business, local community or individual champion that claimed to promote conservation. In six cases (10.5%), the NGO became involved in order to control visitor impacts, after an area of high conservation value became well-known as a tourism destination. For 12 cases (21%), NGOs adopted conservation tourism strategies primarily as a low-impact economic alternative for local communities. In some of these cases, previous industries based on exploiting local natural resources had collapsed through over-exploitation; in some, natural resources had become unavailable through declaration of a new protected area; and in some, there were simply no other resources available for extraction. For 13 cases (22.8%), reasons for NGO engagement in ecotourism were not specified.

### 3.5 Evaluation of outcomes

Case-study authors adopted a wide range of different measures to assess project outcomes: e.g., profits, visitation rates, social benefits, conservation consequences, or changes in environmental policies. Some authors showed that projects may yield social benefits, even where they fail to achieve conservation goals [107, 113]. Some showed that projects may improve the local sense of ownership and environmental awareness, even if they fail to deliver expected economic returns [85, 96, 97, 99].

Few of these case studies, however (S3 Table), quantified the conservation outcomes of NGO-based ecotourism projects accurately or precisely [57, 81, 83, 85, 93–96, 99, 105, 106, 120, 122, 123]. Four mechanisms were identified. Some projects provided direct economic support for reforestation, sustainable agriculture and fishing, garbage collection, and conservation campaigns or lobbying. Some funded wildlife monitoring or patrols in protected areas. Some supported environmental education programs for visitors or residents. A few halted extraction of natural resources from protected areas, by providing an alternative non-extractive livelihood. Case study authors gave little information on the mechanisms used by NGOs to allocate ecotourism profits to conservation efforts, the sums involved, or the tangible conservation gains achieved.

### 3.6 Criteria for success or failure

Criteria put forward by the case-study authors as evidence of project success or failure are summarised in Table 2. They include economic, environmental and sociocultural indicators. For projects judged as successful, case study authors mentioned: economic criteria such as income for local residents; environmental criteria such as support for conservation initiatives, increased conservation awareness, or overcoming conservation threats; and sociocultural criteria such as increased capacity, new skills, increased quality of life, recreational options, social cohesion, political empowerment, protection of culture, and gender equity.

**Table 2:** Criteria used by case-study authors to judge project success or failure.

	Positive benefits	Negative impacts
**Economic**	Employment Economic diversification Sale of other local products Economy dependent on ecotourism Creation of savings, loans, pensions, and other economic compensation	Any income and employment small, insubstantia Only a few inhabitants gain, creating economic inequities Economic inflation
**Environmental**	Support for other conservation initiatives Resources monitoring Promotion of environmental education, increased awareness Halt to uncontrolled development	Exploitation of natural resources through livestock grazing, farming, timber cutting, poaching, hunting, or fisheries; often due to lack of immediate economic benefits from ecotourism Pollution and ecological damage
**Sociocultural**	Increased capacity, training, new skills, new experiences Increased quality of life via new infrastructure and services Promotion of ownership, social cohesion, broader community vision, political effectiveness Protection of culture and promotion of equity	Traditions affected Domestic violence, where men object to involvement of women in projects Feelings of frustration Social division Prostitution and drugs

Where case study authors judged projects as unsuccessful, they commonly cited sociocultural criteria such as conflicts and violence, disorganization and lack of trust, resentment and frustration, prostitution and drug abuse, and damage to local traditions. The projects concerned generated little income or livelihood improvement. Reasons cited include: lack of a competitive tourism attraction; inadequate tourist access and infrastructure; poor marketing and management; and poor quality services provided. Some of these projects hampered conservation rather than enhancing it: e.g., where ecotourism incomes were used for environmentally damaging purchases such as expansion of cattle herds.

### 3.7 Regional signatures

**Fig 2:**
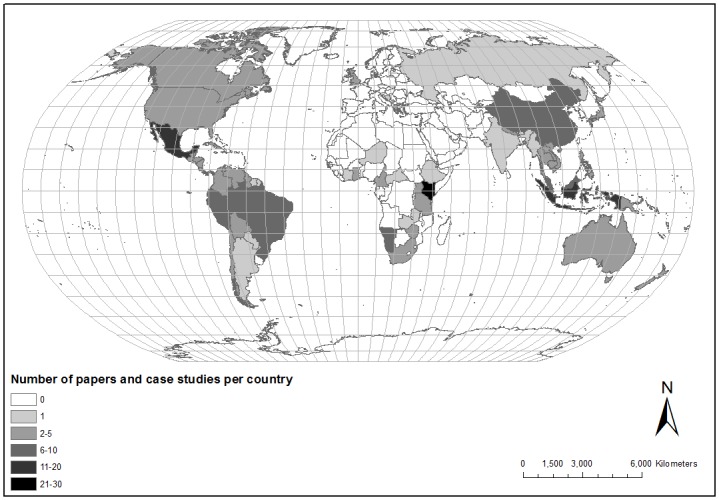
Geographical distribution of case studies. Map created using ArcGIS^®^ (www.esri.com). Published with permission from Esri, under CCAL CC BY 4.0 (http://creativecommons.org/licenses/by/4.0/). Data from this study.

The geographic focus of previous literature is mapped in Fig 2, which contains all the specific cases from S3 and S4 Tables, and the country-specific analyses from S2 Table. Of the 214 specific cases, most (190 studies, 89%) are from developing countries, with a much smaller proportion (24, 11%) from developed countries. Most cases (196, 91%) are located inside protected areas. There is a strong association (p < 0.001) between category and geographic region. In Latin America and Asia, higher proportions of projects were initiated either by NGOs or local communities (categories 3 and 7), whereas in Africa, a higher proportion were initiated by private tour operators (category 9). That is, the predicted regional signatures do indeed exist.

### 3.8 Success factors

Contrary to predictions and suggestions by previous authors [23, 34, 60, 62–65], at global scale we found that the proportions of successful projects is not significantly higher for those led by private operators (category 9) than for those led by other stakeholders (p = 0.52, Fisher’s Exact Test, one-tailed). Whilst many successful private sector conservation tourism projects have indeed been established in Africa during the historical period covered by this analysis (37), only a few involve NGOs (S3 and S4 Tables). In addition, some which do include NGOs have shown limited success [26, 28, 29, 117].

Also contrary to previous suggestions and predictions [32, 66–70], we found that the proportion of successful projects is significantly lower (p = 0.0049) for those which involve international and local NGO’s working jointly, than for those which are operated by a single NGO, either local or international.

## 4. Discussion

This analysis provides the first global-scale review of the role of NGOs in ecotourism. It complements previous reviews of ecotourism in private conservation reserves, communal conservancies, and public protected areas [38, 124]. There are two potential limitations in the scope of projects examined. The first relates to language. Cases from countries such as India, Russia, China or Brazil may be under-represented if they were published in languages other than English or Spanish, unless journals included English titles and abstracts, and are indexed internationally. In practice we identified cases from >70 countries, so it appears that the language limitation is minimal. The second relates to possible publication bias. Case-study authors who report on single-NGO projects may possibly have connections with the NGO’s concerned, and may therefore select more successful projects to report.

This analysis shows that NGOs do indeed play a significant role worldwide in the use of ecotourism for conservation, particularly through community-based approaches associated with protected areas in developing countries. It demonstrates that: there are multiple different mechanisms by which NGOs may become involved in ecotourism initiatives intended to benefit conservation; different NGOs may prefer different approaches; the historical frequencies of different approaches, involving different stakeholders, have differed between continents; and these frequencies may currently be changing, so as to bring greater coherence in practices worldwide. These findings indicate that the paucity of knowledge on the role of NGOs represents a significant gap in current research on ecotourism, community-based tourism and conservation tourism, and merits greater attention.

This analysis also confirms the importance of the relationships between stakeholders for project structure. The stakeholder that first proposes or establishes each new ecotourism project seems to become dominant in determining its initial goals, design, structure, governance and management. Projects proposed by NGOs adopt different management structures and different NGO roles, from projects proposed or established by local communities, private tour operators, or government agencies. This applies even if all the same stakeholders are involved. Some NGOs may later expand or change their roles, even in projects that they did not initiate. Since this may affect project outcomes, it would be valuable to examine in detail what factors promote such flexibility.

The high proportion of cases in developing countries suggests that in these countries, rather than relying on national governments, NGOs endeavour to undertake conservation activities directly, including community engagement and revenue generation through ecotourism [41, 42, 60]. This parallels the approach taken by development NGOs in using tourism for poverty alleviation [23, 43, 57, 60]. In developed nations, where government agencies have more powerful executive functions, NGOs seem to focus instead on political lobbying and formal submissions to public consultation processes [41, 42].

The preponderance of privately-driven projects in Africa, and NGO and community-driven projects in Latin America and Asia, for example, may reflect political histories [78, 97, 125]. In particular, some Asian and Latin American nations have past or present governments whose political ideologies may have created barriers to private sector involvement. In countries such as China, however, this is now changing rapidly, with public-private partnerships widespread for tourism infrastructure in national parks and forests [126]. Some African nations have stable governments and a long history of safari tourism and game lodges, allowing tourism operators to form partnerships directly with local communities [38]. It is also possible that in Latin America and Asia, the use of ecotourism in conservation was started historically by NGO’s, with later involvement by the private sector; whereas in Africa, it was started by private operators, with NGOs becoming involved only recently.

Our analysis shows that historically and at global scale, collaboration between the private tourism sector and NGOs has not increased the proportion of successful ecotourism projects. It also indicates that the proportion of successful projects is greater for NGOs working individually than for those collaborating with other NGOs. There are however, two caveats. The first is lack of detail on the roles adopted by different stakeholders. The second is that local-scale patterns may be masked at global scale. Finer-scale research could examine: how stakeholder perceptions of each other influence partnerships [41]; conflicts between the goals of international and local NGOs [22, 127]; and what drives competing NGOs to cooperate with each other, or not [41]. Some authors, for example [34, 54, 56, 65], have argued that international NGOs have used ecotourism to promote neoliberal political agendas which do not necessarily reflect local concerns.

This analysis reveals that tangible contributions to conservation are rarely quantified in any reliable manner. For example, very few of these 214 studies have measured or reported: contributions to the viability of threatened species populations [128–131]; increases in the number of individuals or area of habitat protected, for threatened species; or reductions in poaching, hunting harvesting, livestock pasturing and land clearing. More accurate and reliable metrics are needed evaluate the environmental outcomes of ecotourism projects involving NGOs. For many projects, NGOs did not articulate conservation goals, nor how they expected to achieve them. Some NGOs may be driven by a desire to conserve particular species, ecosystems or areas; but others by the availability of funding [29, 42].

Improved evaluation of environmental, sociocultural and economic outcomes, and factors contributing to the success or failure of individual projects, is increasingly important as aggregate global investment in ecotourism by NGOs and their donors is now in the tens or hundreds of millions of dollars across the globe [23, 47, 63, 81, 82, 87, 105, 132–136], and investors expect accountability and efficient allocation of resources [34, 137]. Numerous authors have proposed parameters, criteria or approaches to measure the success or failure of individual ecotourism projects [32, 105, 128, 138–142]. It would be valuable to combine or select from these parameters and criteria, and to test them robustly, so as to develop metrics widely applicable under changing global, national, local and internal factors shaping dynamics of the NGOs involved.

The global framework and meta-analysis presented here provides a robust, comprehensive and coherent picture of patterns at global and continental scales, but a coarse-grained one. Many of the individual case studies cited here did provide detailed analyses, but at single-site scales. The more general of the previously published studies identified broad issues and discourses, but with limited detail. We suggest, therefore, that the next step in analysing the use of ecotourism as a conservation tool by NGOs, and the role of NGOs in conservation tourism projects, should be to identify drivers, decision-making processes, designs, and management strategies used across suites of similar initiatives in individual nations or otherwise similar social, political and historical frameworks. We suggest that a political ecology framework could be used to examine how historical processes, cultural factors, actors, institutions and biophysical environments [143–146] combine to determine the drivers and shape the structures and outcomes of ecotourism projects involving NGOs.

In particular, it is noteworthy that whilst four fifths of these cases involved local communities, only one fifth were initiated by these communities. That is, most community-based conservation ecotourism projects are initiated by NGOs. Therefore, the processes which determine how NGOs select and define these initiatives are key in the use of ecotourism in conservation and community development, and deserve future research attention.

## Supporting Information

S1 Table PRISMA Checklist. (DOC)

S2 Table Publications examining the broad roles and approaches of NGOs in ecotourism. (DOCX)

S3 Table Publications describing individual NGO-based ecotourism case studies. (DOCX)

S4 Table Case studies from three compendia (Buckley, 2003, Buckley, 2010, Zeppel, 2006). (DOCX)
