# Supplementary material for: NGO Partnerships in Using Ecotourism for Conservation: Systematic Review and Meta-Analysis
Source: PLoS One. 2016 Nov 28;11(11):e0166919. doi: 10.1371/journal.pone.0166919 (PMC5125656; doi:10.1371/journal.pone.0166919)
Supplement: S2 Table — (DOCX) [file pone.0166919.s002.docx]

S1 Table. Publications examining the broad roles and approaches of NGOs in ecotourism.

| **Country or region** | **Author(s), year** | **Topic analysed and roles of NGO presented** |
| --- | --- | --- |
| Africa, Asia, Latin America | [23] Kennedy K, Dornan DA; 2009 | Various roles and approaches by NGOs using tourism for poverty alleviation in developing countries  Lobbyist, promoter, ongoing manager |
| Alps Region | [51] Schelhaas M,et al.; 2007 | An overview of collaborative actions by NGOs in sustainable tourism in Europe parks  Lobbyist, land owner, land manager, ongoing manager, certifier |
| Argentina, New Zealand | [52] Raymond E; 2008 | Role of NGOs in voluntourism, and its impacts in local communities.  Ongoing manager |
| Australia, UK and USA | [24] Ong F, et al.; 2011 | Guiding considerations influencing voluntourism programs, design of programs and tours, and impacts of tourism from developed countries on local communities in developing countries  Ongoing manager |
| Bosnia-Herzegovina | [36] Karkut J; 1999 | Role of NGOs in a new tourism sector in Bosnia. How NGOs started operations, evolved, and oppose unsustainable tourism developments  Lobbyist, promoter |
| Canada | [40] Lovelock B; 2002 | Relationship between NGOs and government agencies, to manage parks and incorporate tourism components in policy  Lobbyist, promoter, land owner, land manager |
| Cuba | [53] Spencer R; 2008 | Role of NGOs operating cultural tours  Ongoing manager |
| Global | [31] Butcher J; 2007 | Role of WWF, CI, SNV and Tourism Concern in ecotourism and development  Various roles and approaches discussed |
| Global | [57] Hummel J, van der Duim R; 2012 | Describes the role of Netherlands Development Organisation (SNV) in tourism, and its evolution according to global discourses and interests  Various roles and approaches discussed |
| Global | [56] Neves K; 2010 | Discourses by NGOs promoting ecotourism-related activities for whale watching, compare with impacts of whale hunting  Promoter |
| Global | [22] Scheyvens R; 2002 | Various roles of NGOs in tourism and development |
| Global | [34] Simpson MC; 2008 | Identifies stakeholders involved in ecotourism: local communities, government, NGOs and private sector. Discusses problems associated with community-based ecotourism, and calls for detailed analysis of each stakeholder’s role |
| Global | [46] Wearing S, et al.; 2005 | Discusses how NGOs adopt specific strategies according to feminist, ecocentrist, community development and post-structuralist frameworks |
| Global | [33] Wood ME; 1999 | Actions of The Ecotourism Society (TES) in influencing policy makers, developers and in establishing NGO programs  Lobbyist, promoter |
| Guatemala | [54] Sundberg J; 1998 | Argues that predefined agendas and actions by NGOs affect local communities, and are reflected in the landscape |
| India | [50] Singh S; 1999 | Role of NGOs in India in respond to sustainable tourism  Lobbyist, promoter |
| Indonesia | [48] Warren C; 2005 | Under new policies of land and resources use by local communities, NGOs play a significant role in promoting participatory processes for conservation, including ecotourism, which may improve relationship between local institutions and communities |
| Kenya | [43] Barnett T; 2008 | Evolution of Tourism Concern  Lobbyist, promoter |
| Kenya | [27] Lamers M, et al.; 2014 | Stability and governance of tourism partnerships, internal dynamics and external challenges  Broker |
| Kenya | [28] Lamers M, et al.; 2015 | Context influencing and shaping the establishment of ecotourism and arrangements among stakeholders  Broker |
| Kenya | [59] Manyara G, et al.; 2006 | Perceptions of NGOs of local communities in ecotourism initiatives |
| Kenya | [29] van Wijk J, et al.; 2015 | Role of African Wildlife Foundation (AWF) in promoting conservation tourism, through an enterprise model  Broker |
| Lao PDR | [60] Harrison D, Schipani S; 2007 | Dynamics between NGOs, local communities and the private sector under a challenging context  Broker, facilitator |
| Namibia | [35] Hoole AF; 2009 | Partnerships among stakeholders important for success of projects. Cross-scale and cross-level linkages of actors involved  Broker |
| Namibia | [25] Pellis A, et al.; 2011 | Political arrangements, discourses and conflicts of interest among stakeholders  Broker |
| New Zealand | [41] Lovelock B; 2005 | Comparison of two similar collaborative dynamics between government agencies and NGOs in policy-making, park management and tourism  Lobbyist, promoter, land owner, land manager |
| Papua New Guinea | [61] Wearing S, McDonald M; 2002 | Framework to re-think the relationship between tour operators and development agents as intermediaries in rural communities  Broker |
| Paraguay | [32] Jamal T, et al.; 2007 | Links and support from Northern NGOs to small local NGOs, for park management, in a newly democratic nation  Land manager, champion |
| Peru | [55] Valcuende del Río J, Quispe C; 2005 | Conflicts arising after NGO introduced ecotourism without a strategic plan  Champion, facilitator, advisor |
| Southern Africa | [58] Sithole E; 2005 | Government actions, programs and decisions shaping NGO strategies and changing initial conservation goals |
| South Asia, South East Asia and Oceania regions | [41] Lovelock B; 2003 | Perceptions of NGOs in developing and developed nations, related to how ecotourism can support conservation |
| Sub-Saharan Africa | [30] van Wijk J, et al.; 2015 | Historical evolution of African Wildlife Foundation (AWF) in conservation tourism strategies  Broker |
| Uganda | [26] Ahebwa WM, et al.; 2012 | Partnership arrangement affected by complex political and contextual factors, resulting in escalating conflict up to the national level  Champion, advisor, facilitator, consultant |
| UK | [49] Cousins J; 2007 | Role of NGOs as tour operators promoting conservation in developed countries  Ongoing manager |
