# Supplementary material for: NGO Partnerships in Using Ecotourism for Conservation: Systematic Review and Meta-Analysis
Source: PLoS One. 2016 Nov 28;11(11):e0166919. doi: 10.1371/journal.pone.0166919 (PMC5125656; doi:10.1371/journal.pone.0166919)
Supplement: S3 Table — (DOCX) [file pone.0166919.s003.docx]

S2 Table. Publications describing individual NGO-based ecotourism case studies.

| **Country** | **Author(s), year** | **Project name or region** |
| --- | --- | --- |
| Belize | [95] Timothy DJ, White K; 1999 | Toledo Ecotourism Association |
| Belize, Indonesia, Ecuador, Canada | [74, 75] Halpenny EA; 2001, 2003 | Belize and Indonesia: entrance fee scheme for National Parks; Smart Voyager Certification; Group of Research and Education on Marine Mammals (GREMM); Quebec Labrador Foundation, The French Shore |
| Bolivia | [81] Jamal T, Stronza A; 2009 | Chalalan Ecolodge |
| Brazil | [87] Pegas F, Stronza A; 2010 | Brazilian Sea Turtle Conservation Program, TAMAR |
| Brazil | [100] Zanotti L, Chernela J; 2008 | Kayapo, the Brazilian Amazon, pilot project |
| Brazil, Peru | [93] Stronza A, Pegas F; 2008 | Brazilian Sea Turtle Conservation Program, TAMAR; Posada Amazonas |
| Cambodia | [88] Reimer JK, Walter P; 2013 | Chipat Community-based Ecotourism Project |
| Cambodia, Thailand | [98] Walter PG, Reimer J, 2012 | Chambok Community-based Ecotourism Project; Koh Yao Noi Project |
| Chile | [84] McAlpin M; 2008 | Mapu Lahual Network of Indigenous Parks |
| China | [102] Zhuang H, et al.; 2011 | Shitou Township, Yunnan Travel |
| Costa Rica | [73] Gray NJ, Campbell LM; 2007 | Asociacion ANAI |
| Costa Rica | [96] Trejos B, Chiang LHN; 2009 | Women's Ecotourism Association of Chira or AEDC (Asociacion Ecoturistica Damas de Chira) |
| Costa Rica | [44] Trejos B, et al.; 2008 | Costa Rican Community-based Rural Tourism Association, ACTUAR; Consortium of Rural Tourism Co-operatives, COOPRENA |
| Costa Rica | [45] Trejos B, et al., 2008 | Costa Rican Community-based Rural Tourism Association, ACTUAR |
| Costa Rica, Panama | [70] Cusack D, Dixon L; 2006 | The Ngöbe-Bugle community project; APROMOVEN in Las Delicias; MONSELVA Ngöbe and Palo Seco Forest Reserve partnership; FUNDICCEP women’s project; Teribe, Solon and Wetzo; BriBri project |
| Cuba | [92] Spencer R; 2010 | Oxfam Community Aid Abroad Tours, OCAA Tours; Global Exchange's Reality Tours, GERT |
| El Salvador, Zimbabwe | [63] Balint PJ; 2006 | Community-based Ecotourism at El Imposible National Park; Mahenye Safari Lodge |
| Greece | [94] Svoronou E, Holden A; 2005 | Dadia-Lefkimi-Soufli Forest Reserve |
| Honduras | [79] Horochowski K, Moisey R; 1999 | Cuero y Salado Wildlife Reserve; Guaimoreto Lagoon Wildlife Reserve |
| Japan | [78] Hiwasaki L; 2006 | Ogasawara National Park; Kirishima-Yaku National Park; Nikko National Park |
| Kenya | [105] Lamers et al., 2014 | Koija Starbeds Ecolodge |
| Kenya | [103] Nthiga R, et al.; 2011 | Koija Starbeds Ecolodge |
| Kenya | [104] Nthiga R, et al.; 2015 | Koija Starbeds Ecolodge; Sanctuary at Ol Lentille |
| Lao PDR, Bhutan, Vietnam | [80] Hummel J, et al.; 2012 | Ecotourism initiatives at Luang Prabang; Nabji; Sapa District |
| Malaysia | [79] Hamzah A, Mohamad NH; 2012 | Miso Walai Homestay |
| Malaysia | [77] Hitchner SL, et al.; 2009 | Heart of Borneo Conservation Initiative |
| Mexico | [62] Azcarate MC; 2010 | Community-based Ecotourism at Biosphere Reserve Ria Celestun |
| Mexico | [65] Barkin D; 2003 | Community-based Ecotourism at Monarch Butterfly Biosphere Reserve |
| Mexico | [66] Barkin D, Bouchez CP; 2002 | Community-based Ecotourism at Huatulco National Park |
| Mexico | [67] Brenner L, Job H; 2006 | Community-based Ecotourism at Monarch Butterfly Biosphere Reserve |
| Mexico | [72] Foucat VSA; 2002 | Cooperativa de Servicios Ecoturisticos la Ventanilla S.C de R.L |
| Mexico | [89] Segrado R, Farmer F; 2006 | Sustainable Tourism Initiatives in Valle de Bravo |
| Namibia | [82] Lapeyre R; 2010 | Daureb Mountain Guides (DMG) Enterprise |
| Namibia | [85] Novelli M, Gebhardt K; 2007 | Namibia Community-Based Tourism Association, NACOBTA |
| Namibia | [90] Snyman S; 2012 | Torra Conservancy and Damaraland Camp partnership |
| Namibia | [108] Ndlovu J, et al.; 2014 | #Khoadi//Hôas Conservancy |
| Nepal | [64] Baral N, Stern MJ; 2011 | Annapurna Conservation Area Project |
| Nepal | [91] Sparrowhawk J, Holden A; 1999 | Annapurna Conservation Area Project |
| Nicaragua | [71] Somarriba-Chang M, Gunnarsdotter Y; 2012 | The Nature Reserves of Mombacho Volcano and Datanli-El Diablo |
| Nicaragua | [101] Zapata MJ, et al.; 2011 | Renitural |
| Papua New Guinea | [99] West P, Carrier J; 2004 | Community-based Ecotourism at Crater Mountain |
| Peru | [69] Brightsmith DJ; 2008 | Tambopata Macaw Project |
| Peru | [68] Brightsmith DJ, et al.; 2008 | Tambopata Macaw Project |
| Peru | [86] Ohl-Schacherer J, et al.; 2008 | Casa Matsiguenka |
| Saint Lucia | [83] Liburd JJ; 2004 | Aupicon Agriculture and Charcoal Producers and Ecotourism Group, AACP Group |
| Thailand | [97] Walter P; 2009 | Koh Yao Noi Community-based Ecotourism Project |
| Uganda | [107] van der Duim R, et al.; 2014 | Gorilla Tourism in Bwindi Impenetrable National Park |
| Uganda | [106] Nyakaana J, Ahebwa W; 2011 | Kibale Association for Rural and Environmental Development (KAFRED) |
| Zimbabwe | [109] Balint PJ, Mashinya J; 2006 | Mahenye Safari Lodge |
