# Supplementary material for: NGO Partnerships in Using Ecotourism for Conservation: Systematic Review and Meta-Analysis
Source: PLoS One. 2016 Nov 28;11(11):e0166919. doi: 10.1371/journal.pone.0166919 (PMC5125656; doi:10.1371/journal.pone.0166919)
Supplement: S4 Table — (DOCX) [file pone.0166919.s004.docx]

**S3 Table. Case studies from three compendia (**[**37**](#_ENREF_37)**,** [**38**](#_ENREF_38)**,** [**47**](#_ENREF_47)**).**

| **Continent, Country** | **Project’s name** |
| --- | --- |
| ***Africa*** |  |
| Botswana | Xai-Xai Village |
| Botswana | Dqae Qare Game Farm / Reserve |
| Botswana | Controlled Hunting Area KD1 |
| Botswana | Sankuyo Village |
| Botswana | Santawani Lodge |
| Botswana | Gudigwa Camp |
| Cameroon | Mount Cameroon Ecotourism Organisation, MCEO |
| Cameroon | Ebodje Ecotourism Project |
| Cameroon | Lowland Gorilla Ecotourism |
| Central African Republic | Lowland Gorilla Ecotourism Projects |
| Ethiopia | Bishangari Nature Reserve & Lodge |
| Gambia | Tumani Tenda Ecocamp |
| Ghana | Ghana Community Based Ecotourism Project, CBEP |
| Ghana | Amansuri Wetlands |
| Ghana | Tafi Atome Monkey Sanctuary |
| Ghana | Wechiau Community Hippo Sanctuary |
| Ghana | Kakum Canopy Walkway |
| Ivory Coast | Ecotel Touraco |
| Kenya | II Ngwesi Lodge |
| Kenya | Sarara Camp |
| Kenya | Shompole Lodge |
| Kenya | Porini Ecotourism |
| Kenya | Campi ya Kanzi, Kuku Group Ranch |
| Kenya | Kimana Community Wildlife Sanctuary |
| Kenya | Wildlife Tourism on Masai Mara Group Ranches |
| Kenya | Masai Trails & the Olarro Lodge |
| Kenya | Kijabe Ecotourism Project & Kalacha Camp |
| Kenya | CORE Community Ecotourism Ventures |
| Kenya | Mwaluganje Elephant Sanctuary |
| Kenya | Kaya Kinondo Ecotourism Project |
| Namibia | Spitzkoppe Community Rest Camp |
| Namibia | Nyae Nyae Conservancy |
| Namibia | Caprivi Strip Ecotourism |
| Namibia | Bwabwata and Bagani, West Caprivi |
| Niger | Koure Giraffe Tourism |
| Seychelles | Cousin Island |
| South Africa | Dlinza Forest Aerial Boardwalk |
| South Africa | KwaZulu-Natal Conservation Service |
| South Africa | Phinda Private Game Reserve |
| South Africa | Amadiba Adventures |
| South Africa | Ngala Lodge & Game Reserve |
| Tanzania | Jozani Forest & Jozani-Chwaka Bay Conservation Area |
| Tanzania | Manyara Ranch |
| Tanzania | Selous Game Reserve |
| Tanzania | Tanzania Cultural Tourism Coordination Office |
| Tanzania (Zanzibar) | Menai Bay Conservation Area |
| Tanzania (Zanzibar) | Mnemba Island Lodge |
| Tanzania (Zanzibar) | Misali Island Conservation Association |
| Uganda | Mountain Gorilla Tourism |
| Zambia | ADMADE |
| Zimbabwe | CAMPFIRE project |
| Zimbabwe | Vhimba Wilderness Area |
| ***Asia Pacific*** |  |
| Cambodia | Cambodia Community-based Ecotourism Network |
| Cambodia | Yeak Laom Ecotourism Project |
| China | Northwest Yunnan Ecotourism Association |
| China | Wenhai Ecolodge |
| China | Sustainable Tourism in Shangri-La |
| China | Khampa Caravan, Adventure Tour Company |
| China | Jisha Village Ecocultural Tourism |
| China | Wanglang Nature Reserve |
| China | Earth Science Expeditions |
| Fiji | Fiji Ecotourism Association |
| Fiji | Abaca Park & Ecotourism Cooperative Society |
| Fiji | Village-Based Ecotourism Projects |
| Indonesia | The Indonesian Ecotourism Centre |
| Indonesia | Gunung Halimun Ecotourism Enterprise Development Project |
| Indonesia | Rinjani Trek Ecotourism Program |
| Indonesia | Togean Ecotourism Network |
| Indonesia | Operation Wallacea |
| Indonesia | Kandora Mountain Lodge |
| Indonesia | Mentawai Cultural Ecotourism |
| Indonesia | Kayan Mentarang National Park |
| Indonesia | Bina Swadaya Tours |
| Laos | Nam Ha Ecotourism Project |
| Malaysia | Orang Asli Ecotourism & Semelai Community-based Ecotourism |
| Malaysia | Semai Ecotourism & Rafflesia Flowers |
| Malaysia | Sukau Rainforest Lodge |
| Malaysia | Sabah Homestay Programme |
| Micronesia | Utwe-Walung Conservation Area |
| Micronesia | Lenger Marine Protected Area |
| Micronesia | Tamilyong Stone Path |
| Micronesia, Solomon Islands | Pohnpei & Arnavon Conservation Areas |
| Nepal | Kathmandu Environmental Education Project |
| Nepal | Upper Mustang Conservation and Development Project |
| Nepal | Sagarmatha National Park |
| Pacific Islands | Biodiversity Conservation Network |
| Papua New Guinea | Lakekamu, Kaoro Lodge |
| Papua New Guinea | Milne Bay Project, Napatana Lodge |
| Philippines | Mt Pinatubo Trek |
| Philippines | Ifugao Rice Terraces |
| Philippines | Suba Olango Ecotourism Cooperative |
| Philippines | Noslek Arbor Canopy Walk |
| Samoa | Falealupo Canopy Walkway |
| Samoa | Beach *fales* ecotourism |
| Samoa | Tafua Canopy Walkway |
| Solomon Islands | Makira Island Ecotrek |
| Solomon Islands | Rapita Lodge |
| Solomon Islands | Komarindi Ecotours |
| Solomon Islands | Solomon Village Stay |
| Thailand | Hilltribe Trekking |
| Vanuatu | Vanuatu Islands Bungalow & Tourism Association |
| Vanuatu | Aelan Walkabaot Long Vanuatu |
| Vanuatu | Vatthe Lodge |
| Various countries | South Pacific Biodiversity Conservation Programme |
| Vietnam | Be Be National Park Ecotourism |
| Vietnam | Cuc Phuong National Park Ecotourism |
| Vietnam | Hoang Lien Son National Park Ecotourism |
| ***Australia*** |  |
| Australia | Earth Sanctuaries Ltd, ESL |
| Australia | Broome Bird Observatory |
| Australia | Reef Biosearch |
| Australia | ATCV and Nomad Backpackers |
| ***Europe, North America*** |  |
| Canada | Redberry Pelican Project |
| Greece | Prespa Project |
| Russia | Dersu Uzala Ecotourism Development Fund |
| United States | The National Watchable Wildlife Program |
| ***Latin America*** |  |
| Belize | Chaa Creek |
| Belize | Community Baboon Sanctuary |
| Belize | Cockscomb Basin Wildlife Sanctuary |
| Bolivia | Mapajo Ecolodge |
| Bolivia | Agua Blanca Lodge & Lagunilla Lodge |
| Brazil | Amazonian Ecotourism Exchange |
| Brazil | Tataquara Lodge |
| Brazil | Fazenda Rio Negro |
| Brazil | Una Ecopark |
| Chile | Mapuche Ethnic Tourism |
| Colombia | Amacayacu Park |
| Colombia | Kai Ecotravel |
| Dominica | Carib Territory Reserve |
| Ecuador | Napo Wildlife Centre, Amazanga & Sani Lodge |
| Ecuador | Indigenous Network of Alto Napo Communities for the Intercultural Exchange and Ecotourism, RICANCIE |
| Ecuador | Yachana Lodge |
| Ecuador | Kapawi Lodge |
| Ecuador | Galapagos Island |
| French Guiana | Amana Nature Reserve |
| Guatemala | Ecomaya |
| Guatemala | San Andres and San Jose Spanish Schools |
| Guatemala | Conservation Tours Tikal |
| Guyana | Project Guyana, Benab Ecolodge |
| Guyana | Shell Beach Adventures |
| Honduras | Tela Ecotourism Project |
| Mexico | Punta Laguna Ecotourism Project, Najil Tucha Cooperative |
| Mexico | Community Tours Sian Ka'an |
| Mexico | Mots Maya |
| Mexico | Chol Ecotourism |
| Mexico | Commiunity-based Ecotourism Singayta |
| Mexico | Cuatro Cienegas |
| Nicaragua | Community Ecotourism at Pear Lagoon |
| Panama | Pemasky Project |
| Panama | Embera Cultural Ecotourism |
| Panama | Wekso Ecolodge |
| Peru | Machiguenga Centre for Tropical Research |
| Peru | Health River Wildlife Center |
| Peru | Vicos Farmstay |
| Peru | Humacchuco Homestay Tourism |
| Suriname | The Galibi Nature Reserve |
| Venezuela | Angel-Eco Tours |
| Venezuela | Amazonas Ecotourism |
